# Delineation of Homozygous Variants Associated with Prelingual Sensorineural Hearing Loss in Pakistani Families

**DOI:** 10.3390/genes10121031

**Published:** 2019-12-10

**Authors:** Muhammad Noman, Rafaqat Ishaq, Shazia A. Bukhari, Zubair M. Ahmed, Saima Riazuddin

**Affiliations:** 1 Department of Biochemistry, Government College University, Faisalabad 38000, Pakistan; 2 Department of Otorhinolaryngology Head and Neck Surgery, University of Maryland School of Medicine, Baltimore, MD 21201, USA; 3 University Institute of Biochemistry & Biotechnology, PMAS-Arid Agriculture University, Rawalpindi 46000, Pakistan

**Keywords:** prelingual hearing loss, genetic heterogeneity, whole-exome sequencing, genetic testing, intra-familial heterogeneity

## Abstract

Hearing loss is a genetically heterogeneous disorder affecting approximately 360 million people worldwide and is among the most common sensorineural disorders. Here, we report a genetic analysis of seven large consanguineous families segregating prelingual sensorineural hearing loss. Whole-exome sequencing (WES) revealed seven different pathogenic variants segregating with hearing loss in these families, three novel variants (c.1204G>A, c.322G>T, and c.5587C>T) in *TMPRSS3, ESRRB,* and *OTOF*, and four previously reported variants (c.208C>T, c.6371G>A, c.226G>A, and c.494C>T) in *LRTOMT*, *MYO15A*, *KCNE1*, and *LHFPL5*, respectively. All identified variants had very low frequencies in the control databases and were predicted to have pathogenic effects on the encoded proteins. In addition to being familial, we also found intersibship locus heterogeneity in the evaluated families. The known pathogenic c.226C>T variant identified in *KCNE1* only segregates with the hearing loss phenotype in a subset of affected members of the family GCNF21. This study further highlights the challenges of identifying disease-causing variants for highly heterogeneous disorders and reports the identification of three novel and four previously reported variants in seven known deafness genes.

## 1. Introduction

Hearing requires specialized, intricate structures, including mechanosensory hair cells, support cells, stria vascularis, and spiral ganglion neurons (SGNs), in the inner ear [1]. Inner ear hair cell degeneration is the most common cause of hearing loss (HL). However, HL is an extremely heterogeneous group of disorders with both genetic and nongenetic causes that can occur at any age and degree of severity and that affect 1 in 500 newborns and over 360 million people worldwide [2,3]. Many forms of HL are inherited, and approximately 400 syndromic forms occur with linked medical comorbidities. Nonsyndromic recessively inherited HL (NSRHL) accounts for approximately 77% to 93% of hereditary deafness [4] and is genetically complex. To date, 103 distinct autosomal genetic loci have been linked to NSRHL, and among these, 71 mutated genes have been identified. The functions of *NSRHL* gene-encoded proteins range from transcription factors to actin remodelers and extracellular matrix components [4]. Most NSRHL proteins are widely expressed in diverse organs, and yet their genetic defects often cause isolated HL, specifically affecting the intricate inner ear mechanosensory hair, support cells, stria vascularis, or SGNs [5].

In this study, we identified three novel and four previously reported variants in seven HL genes segregating with prelingual NSRHL in consanguineous Pakistani families. In addition to being familial, we also found inter-sibship locus heterogeneity in the evaluated families, which further highlights the challenges of identifying disease-causing variants for highly heterogeneous disorders.

## 2. Materials and Methods

### 2.1. Subjects and Clinical Evaluation

All procedures followed in this study were approved by Institutional Review Board Committees (HP-00061036) at the University of Maryland School of Medicine, Baltimore, MD, USA, and Government College University, Faisalabad, Pakistan. The tenets of the Declaration of Helsinki for human subjects were followed in this study. Informed written consent was obtained from all investigated individuals prior to inclusion in the study. Detailed interviews were conducted with family members to gather information on family structure, comorbidities, onset of disease, and treatment. Clinical phenotyping was performed through a detailed review of medical history, physical examination, pure tone audiometry, a tandem gait test, a Romberg test, and ophthalmic examination. Also, electrocardiography (ECG) and neurological examination was performed on the affected individuals of family GCNF21.

### 2.2. Whole-Exome Sequencing and Bioinformatic Analyses

Whole-exome sequencing (WES) was used to identify the disease-associated variants in the DNA sample of one affected individual from each family. For WES, the genomic libraries were recovered for exome enrichment by using the Agilent SureSelect Human Expanded All Exon V5 kit and sequenced on an Illumina HiSeq4000 (Illumina, San Diego, CA, USA) with an average of 100X coverage. Data analysis was performed with the Broad Institute’s Genome Analysis Toolkit [6]. Reads were aligned with the Illumina Chastity Filter with the Burrows–Wheeler Aligner [7]. Variant sites were called using the GATK UnifiedGenotyper module. Single nucleotide variant calls were filtered by using the variant quality score recalibration method [6]. Filtration of candidate variants was performed as described previously [8]. Primers for Sanger sequencing were designed using Primer3 (http://bioinfo.ut.ee/primer3-0.4.0/). PCR amplification and DNA sequencing were performed as described previously [9]. The American College of Medical Genetics and Genomics (ACMG) classification of the identified variants was performed using the Varsome (https://varsome.com) and Genetic Variant Interpretation (https://www.medschool.umaryland.edu/Genetic_Variant_Interpretation_Tool1.html/) online tools.

### 2.3. Molecular Modeling

Phyre2 (http://www.sbg.bio.ic.ac.uk/phyre2/html/page.cgi?id=index) and HOPE (https://www3.cmbi.umcn.nl/hope/) prediction programs were used to generate 3D structures of the wild-type and mutant proteins and to explore the effects of identified mutations on the encoded proteins. The UCSF CHIMERA online tool (https://www.cgl.ucsf.edu/chimera/) was used for the visualization and variant impact analyses on protein folding and ionic interactions. Clustal Omega (https://www.ebi.ac.uk/Tools/msa/clustalo/) multiple sequence alignment was used to appraise the evolutionary conservation of the identified variants.

## 3. Results

### 3.1. Clinical Data

**Figure 1:**
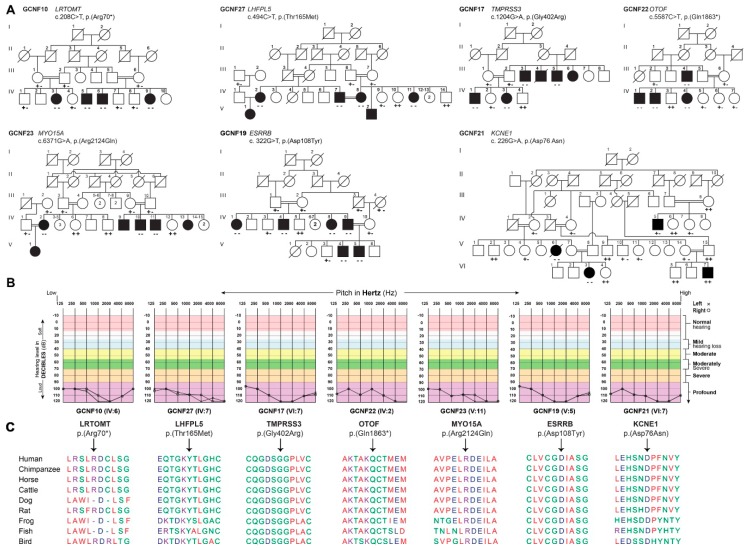
Family pedigrees and hearing loss-causing variants. (**A**) Segregation of disease-causing alleles in seven Pakistani families. Filled and empty symbols represent affected and unaffected individuals, respectively. Double lines indicate consanguineous marriages. The genotypes (wild type and heterozygous and homozygous mutants) of the identified mutant alleles are also shown for each of the participating family members, (**B**) Representative audiometric data from the affected individuals of seven Pakistani families revealed profound hearing loss, (**C**) ClustalW multiple amino acid sequence alignments of orthologous proteins show evolutionarily conserved mutated residues across species.

After Institutional Review Board (IRB) approval and informed consent, seven large consanguineous families (Figure 1A) were enrolled from the Punjab province of Pakistan (Figure 1A). The neonatal clinical records of the affected individuals were not available at the time of enrollment. According to family medical histories, all affected individuals of these families had prelingual hearing impairment. Pure tone audiometric analysis revealed bilateral profound sensorineural hearing loss in all tested affected individuals of these families (Table 1). Romberg and tandem gait tests revealed no obvious balance problems, except in individual IV:3 of family GCNF21. Neurological evaluation revealed epilepsy in two of the affected individuals (IV:5 and VI:7) of family GCNF21. Furthermore, analysis of the electrocardiography (ECG) results obtained from three affected individuals of family GCNF21, using the Schwartz–Moss score method, revealed long QT intervals only in the affected females (VI:3). However, we were not able to perform sophisticated tests of the status of her vestibular system via posturography or a rotary chair. No cornea opacity, peripheral vision, or night blindness problems were noted among the affected individuals. 

### 3.2. Mutation Detection and Molecular Modeling

To determine the genetic causes for nonsyndromic hearing loss segregating in these seven families, whole-exome sequencing was performed for the proband of each family. Autosomal recessive inheritance, both homozygous and compound heterozygous, was assumed during the filtering stages (Table S1). Three novel variants, c.1204G>A, c.322G>T, and c.5587C>T, in *TMPRSS3, ESRRB,* and *OTOF,* and four previously reported variants, c.208C>T, c.6371G>A, c.226G>A, and c.494C>T, in *LRTOMT*, *MYO15A*, *KCNE1*, and *LHFPL5*, respectively, were detected (Figure 1A). Disease-causing variants in these genes have been previously identified in families segregating hearing loss [11,13,14,15,16,17,18,19,20,21]. Variants identified in this study were present in the evolutionarily conserved regions (Figure 1B) of the encoded proteins and were absent or had very low frequencies in the ExAC database (http://exac.broadinstitute.org/) (Table 1). In addition to being familial, we also found inter-sibship locus heterogeneity in our families. The known pathogenic c.226C>T variant identified in *KCNE1* segregates only with the hearing loss phenotype in a subset of affected members of the family GCNF21 (Figure 1A).

**Figure 2:**
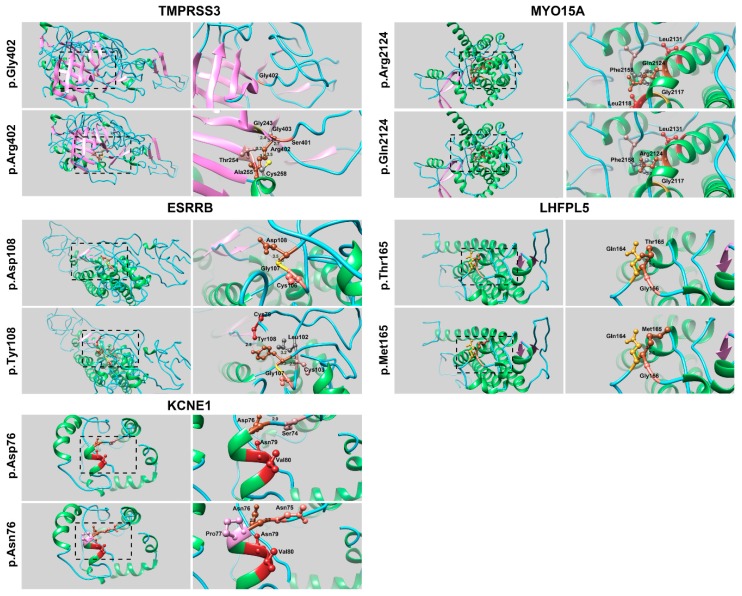
3D structural modeling of human hearing loss-related proteins. Residues of interest are shown in sienna brown for both the wild-type (WT) and mutant amino acids. Red lines represent the hydrogen bonding pattern, while black dotted lines indicate interactions with neighboring amino acids. Different colors are used to show neighboring amino acid interactions. The brick red color indicates the amino acids interacting via hydrogen bonds. Protein helix, strands, and coils are shown in green, pink, and blue, respectively.

To determine the effect of the identified missense variants on the encoded proteins, we performed molecular modeling using Phyre2 software and a number of structures available online at the Protein Database (https://www.ncbi.nlm.nih.gov/protein). The identified variant of TMPRSS3 [p.(Gly402Arg)] in family GCNF17 is present in very close vicinity to the substrate-binding site p.Ser400. Our modeling data suggest that the p.Gly402Arg substitution might result in the loss of correct protein folding, as it introduces a large residue and might add rigidity due to aberrant hydrogen bonds and ionic interactions (Figure 2). Similarly, the p.(Asp108Tyr) variant identified in ESRRB is located next to the zinc-binding site (p.Cys106), and this substitution might introduce new ionic interactions and hydrogen bonding patterns (e.g., with p.Cys79) (Figure 2).

The KCNE1 variant p.(Asn76Asp), identified in family GCNF21, is located within the slow voltage-gated potassium channel domain. The substitution of asparagine with aspartic acid at position 76 results in a charge difference, which does not impact hydrogen bonding (Figure 2) but could impact protein folding and secondary structure. Similar impacts on ionic interactions, hydrogen bonding, or protein folding were predicted for the p.(Arg2124Gln) and p.(Thr165Met) variants found in the MYO15A and LHFPL5 proteins, respectively (Figure 2).

## 4. Discussion

Whole-exome sequencing (WES) has been extremely effective in identifying disease-causing variants for Mendelian disorders, including hearing impairment [22]. Here, using WES, we identified the genetic basis of nonsyndromic hearing impairment segregating in multiple generations of seven large consanguineous Pakistani families. Our study further highlights the genetic heterogeneity of hearing disorders. All seven of these families have variants in seven different known deafness genes, *LRTOMT*, *LHFPL5*, *TMPRSS3*, *OTOF*, *MYO15A*, *ESRRB*, and *KCNE1*.

In two of these families (GCNF10 and GCNF22), nonsense alleles (p.(Arg70*) and p.(Gln1863*)) in LRTOMT and OTOF were found, which truncate the essential encoded protein domains and thus are classified as “pathogenic” according to the American College of Medical Genetics and Genomics/Association for Molecular Pathology (ACMG/AMP) guidelines for the interpretation of sequence variants in known deafness genes (Table 1) [23]. The five missense variants, although predicted to be pathogenic by several in silico tools, have high combined annotation dependent depletion (CADD) scores and are currently grouped into the “uncertain significance” classification (Table 1).

Our molecular modeling revealed a deleterious impact of identified missense variants on the encoded proteins (Figure 2). For instance, *TMPRSS3* encodes a 453 amino acid long type II transmembrane serine protease that plays a vital role in hearing function [17]. The TMPRSS3 variant (p.(Gly402Arg)) found in family GCNF17 is predicted to introduce new hydrogen bonds (Figure 2) that could lead to the loss of serine protease domain activity. Similarly, *ESRRB* encodes a transcription factor that binds with DNA via the estrogen-related receptor (ERR) domain. The ERR domain contains two C4 (four cysteines)-type zinc fingers, each coordinate with one zinc atom. The p.(Asp108Tyr) variant identified in ESRRB in family GCNF19 is present adjacent to the zinc-binding site (p.Cys106) and is predicted to introduce new ionic interactions and hydrogen bonding patterns (e.g., with p.Cys79) (Figure 2). Finally, *MYO15A* encodes an unconventional myosin protein that is known to play an important role in actin organization of cochlear hair cells [24]. The p.(Arg2124Gln) missense variant found in family GCNF23 is located in the MyTH4 (myosin tail homology 4) domain and is predicted to result in the loss of hydrogen bonding with p.Leu2118 (Figure 2). However, these molecular modeling findings warrant functional validation through biochemical and imaging assays.

Similar to our previous studies [25,26], we also found intrafamilial locus and clinical heterogeneity, as some deaf relatives in other branches of family GCNF21 were not segregating the same hearing loss-causal variant (Figure 1). Furthermore, the two affected individuals (V:6,VI:3) homozygous for the variant identified in *KCNE1* also had long QT intervals in addition to hearing loss, while the other two affected individuals (IV:5,VI:5) had epilepsy and deafness. These findings highlight the complexity of the genetic diagnosis of hearing disorder. To facilitate genetic diagnosis in complex consanguineous families, comprehensive clinical phenotyping and subgrouping of affected individuals and exome sequencing of at least one affected individual per sibship/group might be pivotal. 

## Figures and Tables

**Table 1:** Genes, identified variants, and their American College of Medical Genetics and Genomics (ACMG) classification.

Family	Gene	Accession #	CADD	ExAC	cDNA Change	Protein Change	ACMG Classification (Criteria Used)	Reference
GCNF10	*LRTOMT*	NM_001145308	45	0.00	c.208C>T	p.(Arg70*)	Pathogenic (PVS1, PM2, PP3)	[10]
GCNF17	*TMPRSS3*	NM_024022.3	28.6	0.00	c.1204G>A	p.(Gly402Arg)	Likely pathogenic (PM1, PM2, PP2, PP3)	This study
GCNF19	*ESRRB*	NM_004452.3	26	0.00	c.322G>T	p.(Asp108Tyr)	Uncertain significance (PM2, PP3, BP1)	This study
GCNF21	*KCNE1*	NM_001127670.3	15.56	0.00003299	c.226G>A	p.(Asp76Asn)	Likely pathogenic (PS3, PM2, PP3, PP5)	[11]
GCNF22	*OTOF*	NM_194248.3	46	0.00	c.5587C>T	p.(Gln1863*)	Pathogenic (PVS1, PM2, PP3)	This study
GCNF23	*MYO15A*	NM_016239.4	23.8	0.000008398	c.6371G>A	p.(Arg2124Gln)	Uncertain significance (PM1, PM2, PP3, BP1)	[12]
GCNF27	*LHFPL5*	NM_182548.4	29.4	0.00003298	c.494C>T	p.(Thr165Met)	Uncertain significance (PM2, PP3, PP5)	[13]

CADD: combined annotation dependent depletion (https://cadd.gs.washington.edu/); ExAC: Exome Aggregation Consortium (http://exac.broadinstitute.org/); PVS1: pathogenic very strong (null variant (nonsense, frameshift, canonical ±1 or 2 splice sites, initiation codon, single or multiexon deletion) in a gene where loss-of-function (LOF) is a known mechanism of disease)); PS3: pathogenic strong (well-established in vitro or in vivo functional studies supportive of a damaging effect on the gene or gene product); PM1: pathogenic moderate 1 (located in a mutational hot spot and/or critical and well-established functional domain (e.g., active site of an enzyme) without benign variation); PM2: pathogenic moderate 2 (absent from controls (or at extremely low frequency if recessive) in Exome Sequencing Project, 1000 Genomes Project, or Exome Aggregation Consortium); PP2: pathogenic supporting 2 (missense variant in a gene that has a low rate of benign missense variation and in which missense variants are a common mechanism of disease); PP3: pathogenic supporting 3 (multiple lines of computational evidence support a deleterious effect on the gene or gene product (conservation, evolutionary, splicing impact, etc.)); PP5: pathogenic supporting 5 (reputable source recently reports variant as pathogenic, but the evidence is not available to the laboratory to perform an independent evaluation); BP1: benign supporting 1 (missense variant in a gene for which primarily truncating variants are known to cause disease). cDNA: complementary DNA.

## Supplementary Materials

The following are available online at https://www.mdpi.com/2073-4425/10/12/1031/s1: Table S1: Whole-exome sequencing data filtration scheme.
