# Supplementary figures and images for: Delineation of Homozygous Variants Associated with Prelingual Sensorineural Hearing Loss in Pakistani Families
Source: Genes (Basel). 2019 Dec 10;10(12):1031. doi: 10.3390/genes10121031 (PMC6947215; doi:10.3390/genes10121031)

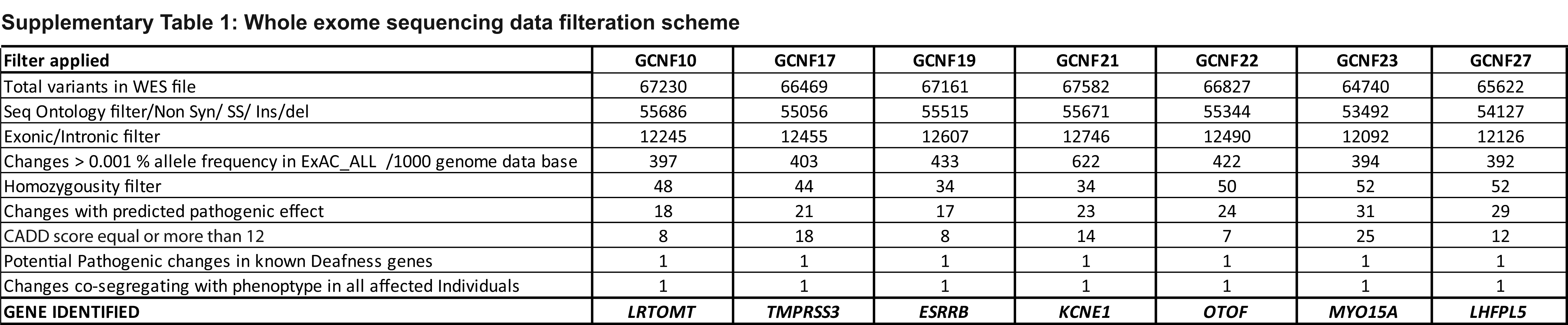

Supplement: Supplementary file 1 [file genes-10-01031-s001.jpg]
